# Food Safety in Home Kitchens: A Synthesis of the Literature

**DOI:** 10.3390/ijerph10094060

**Published:** 2013-09-02

**Authors:** Carol Byrd-Bredbenner, Jacqueline Berning, Jennifer Martin-Biggers, Virginia Quick

**Affiliations:** 1 Department of Nutritional Sciences, Rutgers University, 26 Nichol Avenue, 211 Davison Hall, New Brunswick, NJ 08901, USA; E-Mail: jmartin@aesop.rutgers.edu; 2 Biology Department, University of Colorado at Colorado Springs, 1420 Austin Bluffs Parkway, Colorado Springs, CO 80918, USA; E-Mail: jberning@uccs.edu; 3 *Eunice Kennedy Shriver* National Institute of Child Health and Human Development/National Institutes of Health, 6100 Executive Blvd, Bethesda, MD 20892, USA; E-Mail: quickvm@mail.nih.gov

**Keywords:** food safety, food handling, foodborne illness, consumers, risky

## Abstract

Although foodborne illness is preventable, more than 56,000 people per year become ill in the U.S., creating high economic costs, loss of productivity and reduced quality of life for many. Experts agree that the home is the primary location where foodborne outbreaks occur; however, many consumers do not believe the home to be a risky place. Health care professionals need to be aware of consumers’ food safety attitudes and behaviors in the home and deliver tailored food safety interventions that are theory-based. Thus, the purpose of this paper is to synthesize/summarize the food safety literature by examining the following: consumers’ perceptions and attitudes towards food safety and their susceptibility to foodborne illness in the home, work, and school; common risky food safety practices and barriers to handling food safely; and the application of theory-based food safety interventions. Findings will help healthcare professionals become more aware of consumers’ food safety attitudes and behaviors and serve to inform future food safety interventions.

## 1. Introduction

In recent years, headlines and news flashes on widespread outbreaks of foodborne disease caused by lapses in food safety or emerging pathogens have provided vivid reminders that food not only nourishes and sustains us, but if handled unsafely, can be a major threat to health and well-being. According to FoodNet, the United States’ food safety report card, significant progress had been made toward decreasing foodborne illnesses caused by key pathogens, except Salmonella [1]. This decline is good news, but this rate is still higher than *Healthy People 2020* goals [2] and many people continue to suffer the ill effects of foodborne illness [3,4,5]. Experts estimate that each year 1 in 6 Americans experience foodborne illness—resulting in of the known hospitalizations of 56,000 and death of more than 1,300 [4].

The economic cost of foodborne illness also is high—affecting the U.S. pocketbook at a cost of $50 to $80 billion annually in health care costs, lost productivity, and diminished quality of life [5]. Table 1 displays the estimated total annual economic burden for foodborne illnesses caused by key pathogens in the United States, which account for only about one-fifth of annual foodborne illness cases [5].

**Table 1:** Estimated annual cost per case per year in the United States of selected foodborne illness pathogens *.

Pathogen	Annual Cost Per Case	Annual Number of Cases	Total Annual Cost *
*Bacillus cereus*	$234	63,400	$14,835,600
*Clostridium perfringens*	$482	965,958	$465,591,756
*Norovirus*	$673	5,461,731	$3,675,744,963
*Staphyloccocus aureus*	$695	241,148	$167,597,860
*Campylobacter*	$8,141	845,024	$6,879,340,384
*Shigella*	$9,551	131,254	$1,253,606,954
*Yersinia enterocolitica*	$11,334	97,656	$1,106,833,104
*Salmonella*	$11,086	1,027,561	$11,391,541,246
*Escherichia coli* 0157:H7	$10,048	63,153	$634,561,344
*Toxoplasma gondii*	$39,869	86,686	$3,456,084,134
Hepatitis A	$37,073	1,566	$58,056,318
*Listeria monocytogenes*	$1,282,069	1,591	$2,039,771,779
*Clostridium* *botulinum*	$1,680,903	55	$92,449,665
Total			$31,236,015,107

* Dollar figures derived by multiplying annual number of illnesses by total cost per case [3,4,5].

Many foodborne illness cases and their associated economic costs may be the result of preventable food handling mistakes in the home [6,7,8]. Out of the reported foodborne illness outbreaks reported in the U.S between 1998 and 2008, 9 to 15% were from food-related incidents in the home, with Norovirus and Salmonella being responsible for more than a third of these illnesses [9,10]. Poultry and leafy vegetables are the food vehicle for one in five of these illness-outbreaks [10]. In Europe, approximately one-third of foodborne illness cases were attributed to the home, with half or more of all Salmonella outbreaks traced to the home [11,12]. Because most foodborne illness cases are believed to be sporadic, mild, unconfirmed, and unreported [13], experts estimate that the cases actually originating from food handling errors at home are much higher [6,7,8,14,15,16]—with some estimates reaching 95% [16].

Although experts generally agree that homes are one of the primary locations where most foodborne illness cases occur [14,15,16,17,18,19,20,21,22,23], many consumers do not consider the home to be a risky place with regard to foodborne illness [24]. Almost two-thirds never question whether someone in their household with “flu-like” symptoms (*i.e.*, fever, chills, and nausea) could actually have a foodborne illness caused by foods prepared at home [25,26,27]. In 2011, only 8% of consumers thought the home was a place where foodborne illness was likely to occur, a drop of 10 percentage points since 2005 [28]. Just 12% of consumers believe it is very common for people to get foodborne illness at home [28], and only 7% of those who thought they had had a foodborne illness in the past year reported that home prepared foods were the most likely culprit [29]. Similarly, only 12% of Europeans who suffered a foodborne illness felt it came from home prepared foods [30].

## 2. Why are Homes Such a Risky Place for Foodborne Illness?

There are many reasons why home is the location associated with significant foodborne illness risk. First, the greatest proportion of the food we eat is prepared at home, thereby increasing the opportunities for food handling errors to occur. The emphasis frequently placed on how often people “eat out” causes many to not realize that the home food environment provides 72% of the food, by weight, consumed by Americans and accounts for 93% of the food consumed by those who eat most meals at home [31]. 

Secondly, many people are in groups known to be at increased risk of foodborne illness [2]. For example, 13% of Americans are 65 years and over [32], nearly 7% are less than 5 years old [32], almost 4 million women are pregnant every year [33], and 1% is immunocompromised due to disease, medical treatment, and/or organ transplant [34]. In addition to this, 12 million people are receiving healthcare at home as an extension of or replacement for traditional in-patient care [35]. This amounts to one quarter of the U.S. population being at increased risk for foodborne disease and at high risk for severe health outcomes if they become ill [2]. 

Thirdly, many consumers—even those in high risk groups—do not perceive themselves or someone in their families to be susceptible to foodborne illness [36], rank their risk of foodborne illness lower than that of others [37], or do not follow all recommended food safety practices [38], and consequently they do not take sufficient precautions. For instance, young infants are particularly vulnerable to infections due to their immature immune systems which makes scrupulous cleaning and handling of equipment associated with infant feeding critical [39]. Although women report they become interested in food safety after they have a baby [40], there are numerous documented food handling mistakes with regard to infant feeding [15,41,42,43]. Researchers in the United Kingdom, for example, found that 4% of baby bottles that parents indicated had been cleaned, disinfected, and ready to be filled were actually contaminated with *Staphylococcus aureus* [43]. This contamination is especially problematic given that bottles often are prepared in advance for feeding later; this time lag provides sufficient opportunity for significant pathogen proliferation [43]. Food handling in homes of young children needs improvement given that “children younger than age 4 years have the highest incidence of laboratory-confirmed infections from: *Campylobacter* species, *Cryptosporidium* species, *Salmonella* species, *Shiga* toxin-producing *Escherichia coli* O157, *Shigella* species, and *Yersinia* species” [2]. 

Additionally, food prepared in the home may be served to a wider community, such as at bake sales, church dinners, and school picnics, or even foods children trade at lunchtime or homemade snacks adults share with colleagues in the workplace. Food samples served at farm market stands also are commonly prepared in home kitchens [44]. At least one-fifth of young children in the U.S. also are in home-based (non-parental) child-care where food may be served [45].

Unlike commercial enterprises, home kitchens are multipurpose areas and are much more than just food preparation and storage places [16,22]. For instance, researchers have observed women’s purses that once sat on public ladies’ restroom floors were sitting on kitchen counters [22]. Pets, old newspapers, dirty laundry, house plants, and soil all are common in home kitchens—one research team even reported observing a home kitchen where automotive repairs were occurring [16,22]. Kitchen sinks are used for hand washing, produce washing, dishwashing, soaking clothing, washing children and pets, and wetting mops. Dirty dishes may be stacked alongside clean dishes on kitchen counters. Raw unwashed vegetables, dripping raw meat, as well as cooked ready-to-eat foods are common in home refrigerators. The multiple uses of home kitchens provide risky potential to introduce an array of pathogens that can spread to foods, proliferate, and result in illness. Some of the pathogens that have been confirmed in home kitchens include Salmonella, pathogenic *Escherichia coli*, *S. aureus*, and *Campylobacter* [46,47]. At least two studies have reported that the kitchen is more heavily contaminated with fecal coliforms than bathrooms [47,48].

## 3. What are Consumers Doing with Regard to Food Safety?

Even though food handlers—including home food handlers—are the last line of defense in the food safety chain, most have not had a food safety course recently or at all [49,50,51]. Opportunities for children to learn safe food handling in schools have declined as family and consumer science courses have become less common [51,52]. As a result, many teens and adults have limited food preparation experience, have not learned food safety strategies, and lack the basic knowledge needed to keep themselves and their families safe from foodborne illness [49,52,53,54,55,56]. 

On the positive side, about 6 in 10 consumers recognize they are responsible for food safety and nearly all say they gave at least some thought to food safety in the past year [28,57]. But there are gaps in consumer applications of Clean, Separate, Chill, and Cook advice from the Dietary Guidelines for Americans and food safety programs like *Fight Bac!* and the World Health Organization’s *Five Keys to Safer Food* [22,41,58,59,60,61,62,63,64,65,66], that have been documented in the U.S., Australia, Ireland, Italy, Slovenia, and Turkey [15,67,68,69,70]. Inconsistent practices among home food handlers can negate much of the effort made in improving and maintaining food safety achieved earlier in the food chain [15,52].

## 4. Consumer Food Safety Practices

### 4.1. Clean

The goal of “clean” is to prevent cross contamination—or the transfer of disease causing microorganisms from one food, object, or surface to another food—by washing hands, food contact surfaces, and kitchen equipment [58,66]. Hands are a major “vehicle” for spreading pathogens around the kitchen [71,72]—thus hand washing is critical to preventing cross contamination [73,74].

Almost all consumers report washing their hands with soap for a full 20 s before preparing food all or most of the time [72,75,76]. Most consumers also report they often or always wash their hands after handling raw meat [28,30]. Despite consumers’ awareness of the importance of hand washing, they are not washing their hands thoroughly. For example, after handling raw chicken, 73 to 100% of hands of consumers who reported washing their hands after touching the meat in a research study) were contaminated with *Campylobacter jejuni* [30]. None of the consumers sufficiently washed their hands to prevent *C.* *jejuni* transfer to salads after handling the raw chicken [30]. 

Little is known about how *often* during meal preparation consumers wash their hands. Given how often the most heavily contaminated areas in the kitchen (*i.e.*, refrigerator handles, tea kettle handles, tap handles, sink drain areas, dishcloths, and sponges [16,77,78]) are touched during meal preparation, it is likely that hands are not washed frequently enough to prevent the transfer of pathogens to ready-to-eat food [73,79,80], food packaging, or equipment and contact surfaces used to prepare food [16,81]. Table 2 shows pathogens that have been identified on frequently touched areas of the home kitchen [16,78]. 

**Table 2:** Key pathogens found on frequently touched areas of the home kitchen *.

Site	*Campylobacter*	*Salmonella*	*S. aureus*	*E. coli*	*L. monocytogenes*
Dishcloth, sponge, towel	✓	✓	✓	✓	✓
Sink, tap handles		✓	✓	✓	✓
Refrigerator handle	✓		✓	✓	✓
Trash	✓		✓	✓	
Cutting board	✓		✓	✓	
Work surface	✓		✓	✓	
Floor	✓			✓	

* Adapted from Redmond *et al.* [15] and Griffith [76].

Dishcloths and sponges quickly become heavily contaminated with a diverse array of microbes, harboring and spreading contamination to hands, kitchen equipment, and contact surfaces [16,77,82,83,84,85,86]. High numbers of *E. coli* survive in dishcloths for at least 48 h [79]. Consumers have room for improvement when using sponges and sanitizing dishcloths—of the 92% of consumers who use them, just 9% report changing dishcloths or sponges daily, 44% change them at least weekly, the remainder change them less often, with 5% waiting until they fall apart [75].

Kitchen utensils and cutting boards also are key cross contamination routes [30]. In fact, research in the U.K. suggests that 14% of all foodborne illnesses may be due to inadequately cleaned cutting boards and knives [15]. Although nearly all consumers report they wash these items after using them with raw meat or produce, observational data indicate that the vast majority of consumers do not clean cutting boards and utensils sufficiently to prevent cross contamination [21,59,75,87,88,89]. 

Cleaning of food products prior to consumption and preparation is another important component of “clean”. A recent study recommended that consumers use a 3% hydrogen peroxide solution (which is readily available at pharmacies) to wash cantaloupes prior to cutting [90]. A misperception remains that washing raw poultry removes “germs” [91], hence providing clear and accurate information regarding which food products require washing (and how to properly do so) before preparation is needed. 

A relatively new cross-contamination vehicle in the U.S. that has the potential to pose a significant risk of bacterial cross contamination is reusable grocery bags. One in three consumers report using these bags for more than just groceries [92] —they double as gym bags, toy bags, and other uses. This is a concern given that 75% of consumers use these same bags for carrying raw meat and other foods [92]. Large numbers of bacteria (including fecal coliforms) were found in every reusable bag collected from consumers outside a grocery store, but none were found in new bags or traditional plastic bags [92]. Despite the effectiveness of removing pathogens by washing reusable grocery bags, only 3% of consumers reported regularly washing them [92]. Dirty reusable grocery bags could pose a foodborne illness risk—an outbreak of norovirus in a girls soccer team was traced to a contaminated reusable grocery bag [93].

### 4.2. Separate

“Separate”, like “clean”, is vital to preventing cross contamination. The goal of “separate” is to keep raw meat, poultry, and seafood separate from ready-to-eat foods like salads and cooked meat. About three-quarters of consumers report keeping raw meat, poultry, and seafood separate from ready-to-eat food products and nine in ten use different plates for raw and cooked meat [75]. However, there is room for improvement especially considering that meat, poultry, and seafood are the leading causes of foodborne illness [94,95]. 

### 4.3. Chill

“Chill” focuses on the refrigerator’s critical role in temperature control. But, it is important to also think about “clean” and “separate” in this appliance. Studies indicate that refrigerators in many households are not clean. One study from Ireland reported that more than half of the refrigerators swabbed had at least one of these pathogens: *S. aureus*, *Salmonella enterica*, *E. coli*, *Listeria monocytogenes* and *Yersinia enterocolitica* [15].

Many refrigerators are also not cool enough, with average temperatures exceeding the recommended 5 °C (40 °F). This problem has been noted in the U.S., U.K., Ireland, New Zealand, and Australia [15,16,22,24,96,97,98,99,100]. Compounding the cooling problem is that refrigerators often are packed so tightly with food that air circulation is restricted [22]. Tight packing also increases food-to-food cross-contamination risk [22]. Only one-quarter of consumers report regularly checking refrigerator temperatures, and another quarter do not even have a refrigerator thermometer [15,75]. One positive note is that nearly 60% of those in the U.S. know the safe temperature for refrigerators to be less than 5 °C (40 °F) [75]. 

Another aspect of “chill” is keeping perishable foods out of danger zone temperatures. Most consumers (79%) reported leaving prepared perishable food at room temperature no longer than the recommended two hour timeframe and nearly two-thirds report thawing food in the refrigerator [75]. There also is a common misconception that cooked foods should be cooled to a room temperature before being placed in the refrigerator [101]. 

### 4.4. Cook

The greatest area needing improvement is “cook”—according to Healthy People 2020, only about 37% of consumers achieve the goal of heating foods, such as meat and poultry, to a temperature sufficiently high enough to kill harmful pathogens [2]. Almost 9 in 10 consumers know that ground beef should be cooked to at least 160 °F (71 °C) and nearly two-thirds report they usually cook meats and poultry to this temperature [75]. However, most do not know that color is not a good indicator of doneness [51,102] and less than a quarter actually validate the accuracy of the cooking temperature with a thermometer [75]. Consumers continue to not use thermometers despite knowing that the greatest food poisoning risk is from undercooked foods and that “germs” in food are serious dangers [29,75,88]. 

Many consumers report that thermometers are inconvenient and difficult to use, especially with small cuts of meat, which contributes to low rates of thermometer use [103]. Many express frustration with needing to remember the different temperature recommendations for beef, poultry, and seafood [104]. Also, recipes and cooking shows seldom give endpoint temperatures [105]—recommending color as an indicator of meat doneness three times more often than temperature [106]. Color recommendations and lack of thermometer use in televised cooking shows may lead consumers to view using food thermometers as a sign of being an inexperienced cook [88]. But, visual inspection is risky—for instance, 70% of the chicken pieces consumers judged as “done” by visual inspection had not reached recommended cooking temperature and had active *C. jejuni* cells [72,107]. 

Microwave ovens are nearly ubiquitous in home kitchens throughout the developed world and play an important role in achieving the “cook” goal. Many consumers report that they regularly follow cooking, flipping, rotating, stirring, and standing instructions and check to be sure food is fully heated before eating. However, few use a thermometer to verify food has been sufficiently heated in a microwave [75]. In addition, microwave ovens are seldom cleaned and present a cross-contamination risk [22].

### 4.5. Risky Foods

A fifth category of food safety practices that typically is not part of most food safety campaigns is eating “risky” foods. Substantial proportions of the population consume foods that pose a significant foodborne illness risk, such as undercooked animal flesh. For instance, more than half of U.S. consumers report eating raw cookie dough (made with raw eggs), two-fifths consume raw eggs, a quarter eat raw fish, one-fifth eat undercooked hamburgers, and an eighth eat raw oysters [53,88]. 

### 4.6. Costly Errors

Medeiros and colleagues matched key pathogens with their primary control factor [108] to estimate the cost associated with each type of food handling error (*i.e.*, clean, separate, chill, and cook food handling errors and risky eating behaviors) [5,108]. As shown in Table 3, cook and separate errors are the most expensive at an estimated annual cost of $23.5 billion followed by lapses in personal hygiene at $5 billion [5,108]. These likely are underestimates because only key pathogens were included.

**Table 3:** Foodborne illness cost annually by primary control factor *.

Primary Control Factor	Common Foodborne Pathogens	Annual Cases	Annual Cost
Clean	Norwalk, *Shigella*, Hepatitis A	5.6 million	$5 billion
Chill	*C. perfringens*, *S. aureus, Bacillus cereus*	1.3 million	$649 million
Cook & Separate	*C. jejuni*, *Salmonella*, *Toxoplasma gondii*, *Yersinia enterocolitica*, *E. coli 0157:H7*	2.1 million	$23.5 billion
Avoid Risky Food	*L. monocytogenes*, *C. botulinum*	<2,000	$2.1 billion

* Categorization by primary control factor based on Medeiros *et al.* [105]. Annual cases and costs derived from Scharff [5].

## 5. What about Food Safety at Work and School?

Besides the home, work and school are places where most people spend much of their time. Home prepared meals and snacks are commonly eaten at these places. Many workers eat lunch at their desks. This poses a foodborne illness risk, with only 38% cleaning their work area weekly and a similar proportion of workers report rarely or never cleaning it [75]. Only half of workers report always washing their hands before lunch, and less than a quarter of workplace refrigerators are cleaned weekly [75]. “Chill” practices at work are somewhat better, with two-thirds of workers reporting they store their lunches in the refrigerator, yet half also report leaving perishable foods at room temperature more than two h [75]. 

Lunches children take to school may pose “chill” food safety problems because they are often stored at room temperature for several h and some schools require children to dispose of leftovers and packaging at home [109]. An examination of lunches brought by preschoolers revealed that most (88%) lunches were at room temperature [110]. Less than 2% of lunches containing perishable items were not at danger zone temperatures [110]. Even with multiple ice packs, nearly all lunch items were at unsafe temperatures [110]. A study of the “temperature journey” of packed lunches children took to school in Ireland revealed that lunches were in the danger zone temperature range almost as soon as school began and remained there all day [109].

Reusable lunch bags and boxes create a “clean” food safety hazard as they are frequently cleaned by wiping with a dishcloth [109], one of the most significant vehicles for cross contamination in home kitchen [77,82]. This cleaning method may add pathogens instead of removing them. Zippered, insulated lunch bags cleaned by wiping with a damp cloth have been found to have the highest microbial count, with some testing positive for *S. aureus* [109].

## 6. Who is Most Likely to Make Food Handling Errors?

Those most likely to mishandle food are men, adults younger than age 30 years or older than 64 years, and those who have at least some post-secondary education [21,64,70,87,88,111,112]. According to Fisher and colleagues, “*People who prepare food least frequently…are…the most dangerous cooks*” [112].

However, consumers at all ages make significant mistakes when it comes to handling food safely—even those at increased risk for poor outcomes from foodborne illness [21,23,64,108,113]. For example, some pregnant women have reported they do not refrigerate perishable food within 2 h, use a thermometer to ensure foods reached a safe temperature during cooking, or use safe thawing practices [41,42]. They also report eating soft cheeses and blue-veined cheeses and hot dogs or deli meats without reheating them [41,42]. Some mothers of young babies also report they do not wash hands with soap and water before preparing food, after changing diapers, or before feeding infants [15,41,42].

There is some evidence that certain foodborne illnesses are common in some racial/ethnic groups than others due to cultural difference in food intake and/or handling practices [114]. For instance, Listeriosis incidence is higher among Hispanic women most likely because of the popularity of Mexican-style cheeses [115,116]. Yersiniosis is also thought to be more common among African American infants and children due to a food culture that includes fresh chitterlings (cleaned and cook pig intestines) [117,118]. Compared to minority groups, *E. coli* 0157:H7 infections are more common among Caucasians because of their tendency to eat more raw and undercooked foods [64,119].

## 7. Why is It so Hard to Adopt Safe Food Handling Practices?

There are many reasons consumers may mishandle foods. Some of the more common barriers to safe food handling are described below.

### 7.1. Automatic Pilot

“When I make dinner, I have my routine. I really don’t have to think about it too much.” Food preparation, for many, is a repeated or habitual behavior [112]. The more often a task like preparing food is repeated, the more “automatic” it becomes—that is, less cognitive effort is needed [120,121]. Intervening to break this chain of events by introducing a new procedure (e.g., using soap to wash hands instead of just rinsing them, using a thermometer to judge doneness instead of just assessing color) is challenging. 

### 7.2. Responsibility Deflection

“It’s not my responsibility.” Some consumers feel food safety is the responsibility of others higher in the food safety chain who control food safety risks prior to food being offered for sale [29,122]. As a result, they deem food safety as not important in the home environment [29,109,122], and may not accept their role in preventing foodborne illness in the home [72]. 

### 7.3. Risky Preferences

“I enjoy eggs with a runny center.” “I prefer the taste of rare meat.” These consumers are concerned the new behavior will change the taste of foods and diminish their pleasure [71]. This is important to consider given that taste is the number one driver of food choices [57]. 

### 7.4. Cost: Benefit Miscalculations

“That takes too much time!” “It’s inconvenient.” Some feel the time, effort, and resources needed to make the change are not reasonable or convenient [71,123,124]. Foodborne illness often is mild and of short duration, thus many consumers may not be aware of its sometimes devastating and deadly outcomes when they (mis)calculate the value of safe food handling procedures [62]. 

### 7.5. Social Fears

“What would my family think if I checked their burgers with a thermometer?” Individuals with the primary responsibility for preparing foods in their household indicate that they take great pride in their cooking [125]. In addition, they highly rate the quality of the work done by those who prepare foods in their homes to ensure the safety of their food [57]. Some household food preparers feel that new behaviors, like using thermometers to check cooking temperatures, would diminish the opinions others have of their skills as a cook [126]. 

### 7.6. Faulty Outcome Expectations

“*I’ve always done it this way and haven’t gotten sick.*” These consumers do not perceive that the current way of behaving is problematic (or making them susceptible) to foodborne illness [127]. They may fail to understand how emerging pathogens and changes in the food supply make what was once a safe behavior (e.g., eating raw eggs or rare burgers) a risky behavior. Compounding this problem is that few believe that home prepared foods are a likely cause of foodborne illness [29]. 

### 7.7. Optimistic Bias

“It won’t happen to me.” Nearly 6 out of 10 consumers believe their chances of getting foodborne illness are low [57]. Some consumers believe that they have a small chance of getting a foodborne illness compared to others [57]. This optimistic bias is positively linked with risky behaviors and neglects to take precautionary measures, which is related to increased incidence of accidents and foodborne illness [128,129]. 

Most (90%) report their personal risk of illness from eating food *they* prepared is low [130]. But, when asked about the risk of others in their social group, only 41% thought these individuals had a low risk of illness from eating food they prepared [130]. Rating one’s own risk as lower than others in one’s social group—those with whom an individual compares him or herself—is an indicator of low motivation to change precautionary behaviors [37,129].

### 7.8. Illusions of Control

“We take the necessary precautions in my home.” Two-thirds believe they exert high levels of control over safe food handling when they prepare food [130]. When asked what grade a food safety expert would give them for food preparation, service, and storage in their home, all but 2% gave themselves passing grades [75]. When consumers used a retail food establishment food safety evaluation checklist adapted for homes, scores were considerably lower [131,132]. Scores were even lower when trained auditors evaluated home kitchens—the average grade was failing [22,133,134]. Making these results more disheartening is that participants in these home visit studies were aware that researchers would be coming to their home to observe and evaluate their kitchen practices. 

## 8. How Can Health Professionals Help Consumers Handle Food More Safely?

Health behavior change theories, such as the Health Belief Model, Theory of Planned Behavior, and Social Cognitive Theory, provide valuable roadmaps for identifying key constructs to address when aiming to effect behavior change. Although thousands of studies demonstrate the usefulness of these theories in designing effective interventions for a wide array of health behaviors, including food safety [135], few food safety interventions have been theory based [136,137,138]. Food safety education programs built on the constructs below have the potential to help consumers gain the knowledge, skills, motivation, and confidence needed to handle foods more safely [126,135,136,137,139,140,141].

### 8.1. Boost Knowledge

For behaviors associated with Clean, Separate, Chill, and Cook, many consumers are aware of the food safety basics [53,70,71,123]. Consumers also understand that “germs” can hurt them [28]. However, many still have food safety knowledge gaps and their knowledge of safe food handling practices does not always correspond with reported use [64,70,139]. This suggests a need to build consumer knowledge, activate existing knowledge, and motivate information application.

### 8.2. Highlight Responsibility

Consumers are less likely to take protective steps when consumers place less importance on their own responsibility than that of others in the food safety chain or believe their risk of foodborne illness is controlled by fate or luck [142,143]. Some feel they have little responsibility because they believe most foodborne illnesses are caused earlier in the food safety chain or by retail food establishments [18,62,63]. Helping consumers understand the magnitude of control they have in their own homes as food safety risk managers and finding motivators—such as helping them understand that by using a thermometer, loved ones are less likely become sick from undercooked meat or showing them how easy thermometers are to use—can help promote behavior change [136,139]. 

### 8.3. Heighten Recognition of Susceptibility and Severity of Outcomes

Engaging in health protective behaviors is associated with greater perceived susceptibility or beliefs in the likelihood of a negative health outcome and its severity [127,144,145,146]. For example, those who believe food poisoning is a personal threat eat fewer risky foods [147]. Personalizing risks can help consumers better understand their own foodborne illness susceptibility [127]. Thus, interventions should help consumers learn who is at increased risk for foodborne illness as better knowledge of these groups predicts better compliance with safe food handling recommendations [148]. 

### 8.4. Emphasize Behavioral Control

Perceived behavioral control is a significant predictor of safe food handling intentions [136]. However, it is difficult to motivate consumers to change when they feel confident that they are already controlling foodborne illness risks in their kitchens [127]. Tools that help consumers pinpoint problems in their own kitchen, such as home kitchen food safety self-assessments, could personalize the message and increase their awareness of problem spots [131,132]. These tools also can clarify how current behaviors could be endangering their health and how simple changes can lower the danger level. Another tool is “recipe Hazard Analysis and Critical Control Point (HACCP)”—that is, teaching consumers to identify steps in a recipe that may increase food safety hazards and think ahead about how to resolve them [149].

### 8.5. Build Confidence

If consumers are worried about possible embarrassment of performing new behaviors, like using a cooking thermometer, improving attitudes toward the behavior and changing beliefs about how others in their social network perceive the behavior can build the confidence needed to motivate consumers to make changes [139,150]. Social networks may influence a broad array of health-risk behaviors, especially among adolescents [150,151,152,153,154,155]. Although few studies examining the role of social influences on food safety behaviors could be located [123,126,136,156], studies of other health behaviors strongly suggest that utilizing social networks in food safety interventions could increase their effectiveness [126,150,151,152,153,154,155]. In addition, working to shift social norms, such as by modeling thermometer use or other food safety behaviors on television programs can help build confidence [150,157] (an example of a video clip that shifted social norms that may be familiar to readers is the “double dipping” clip from a Seinfeld episode [158]. 

### 8.6. Offer Cues to Action

Researchers have reported that consumers take food safety precautions only when they perceive a risk, such as when they handle raw poultry, fear they may give others food poisoning, or when others are watching [123,136,159]. At other times, consumers may be acting out of habit and make food handling mistakes because they lack “cues to action” [88,160]. Fein and colleagues use the analogy of driving a car—drivers are constantly taking protective actions in response to cues, such as the yellow stripe in the middle of the road or a stop sign [88]. But, when making dinner, hazards are not visible (e.g., pathogens on the unwashed produce that are contaminating the counter and our hands) and there are few, if any, cues to remind us to practice safe food handling (e.g., use soap to wash hands or keep washed and unwashed produce separated). 

Risk messages or handling instructions on food packages help cue some to change their behavior [161]. In one study, the control group received a chicken salad recipe and the experimental group received the same recipe with a printed message encouraging them to take great care to avoid cross contaminating the salad by preventing raw meat juices from coming in contact with other ingredients and utensils. Salads made by the group receiving the cue had significantly less bacteria than those made by the control group, putting the experimental group at a four-fold lower relative risk of falling ill than the control group [162]. Another study that involved preparing a chicken salad recipe found that only 57% of important hygiene measures (*i.e.*, washing hands with soap and water, checking doneness with a thermometer) were used by participants [163].

Adding food safety cues to food packages may be particularly effective given that nearly half of consumers indicate they commonly read cooking instructions on food packages [57]. Placing soap dispensers in direct line of sight also helps improve hand washing [164]. Adding endpoint cooking temperatures in educational materials and cookbooks are other cues to action [126,139]. Printing washing instructions on reusable grocery bags could cue consumers to wash them [92].

## 9. When and How Do Consumers Want Food Safety Information?

Consumers—from middle schoolers to older adults—are interested in the safety of their food and have a positive attitude toward learning about food safety [76,104,165,166]. Thus, if it is delivered “their way”—that is personalized and tailored to the sensibilities and interests of their sociodemographic group [108,140,166,167,168]—consumers may be amenable to food safety information anytime. Health communications and marketing experts tell us that educational programs and materials directed to “everyone” actually meet the needs of few [49,135,140,169,170,171,172], yet with few exceptions, the target audience of many of the food safety education programs continues to be broadly defined (e.g., American consumers). Creating and delivering personalized, tailored food safety education presents both a challenge and a promise for advancing progress toward food safety goals of *Healthy People 2020* [2] and the food safety global strategy of the World Health Organization [173] at reducing key food handling errors made at home.

Some especially “teachable moments” identified by researchers are after publicized foodborne illness outbreaks or recalls, before major holidays, during the perinatal period, and after being diagnosed with an immune-compromising condition [29,40,88,140,174]. However, providing food safety information for those at increased risk of poor foodborne illness outcome often is not part of standard clinical practice among health professionals [36]. Similarly, athletes seeking sports health advice rarely receive food safety information despite the foodborne illness risk they face in the often long-distance transport to and lengthy unrefrigerated storage of food at training and competition venues (personal communications from J. Berning: Food handling errors of athletes (2012)). Drinking from the same water bottle or other beverage container is another common food safety hazard [49]. 

Of those who eat out, 9 in 10 Americans report they take leftovers home at least occasionally and 32% do so regularly [57]. These consumers are interested in food safety, as three out of four diners feel it would be helpful to receive storage and reheating instructions for leftovers or take out [57]. Despite the potential ease of printing information on take-out food containers, this type of information is rarely included. 

Other frequently missed food safety education opportunities are food stand volunteers at fairs and other community events [175,176], agritourism personnel selling ready-to-eat food along size fresh produce and farm animals [177], and farm market staff offering product samples [178]. Another important missed opportunity is the lack of incorporating food safety concepts in elementary and secondary school science textbooks. Food safety professionals, especially those at under-funded agencies who have not been able to attend professional meetings to gain the latest information regarding food safety training, are themselves among the many professionals needing greater access to advanced training opportunities so they may educate their constituents regarding new and developing issues of food safety [179]. 

Health professionals often recognize consumers’ need for food safety information and feel they have an important role in educating and counseling clients about home food safety, yet food safety typically is not a high priority [75]. Even consumers at high risk of foodborne illnesses may not be offered food safety advice [75]. It is also important for Registered Dietitians to keep in mind myths, misperceptions, and attitudes about safe food handling that may be present in various cultures [91,180].

## 10. Conclusions

Tremendous legislative, agricultural, industrial, and public health efforts have been devoted to improving the safety of the food supply, but these efforts are in vain if not matched by safe food handling at home [181]. Reaching and engaging consumers is challenging because they have many competing interests, may not see the inherent value of food safety education, have misinformation or misperceptions about safe food handling, and engage in culturally-driven food handling practices that are at odds with current food safety recommendations [49,91,180]. However, the high rate and cost of foodborne illness highlights the need for health professionals to develop and implement more effective (*i.e.*, behaviorally focused, theory-driven, tailored, and personalized) food safety educational programs that result in safer food handling practices of consumers at all ages [108,138,168,169,182,183]. Clearly, there are many opportunities for health professionals to extend their practice by incorporating safe food handling in consumer communications. 
